# Naturally infected *Aedes aegypti* collected during a Zika virus outbreak have viral titres consistent with transmission

**DOI:** 10.1080/22221751.2018.1561157

**Published:** 2019-02-04

**Authors:** Sasha R. Azar, Esteban E. Diaz-Gonzalez, Rogelio Danis-Lonzano, Ildefonso Fernandez-Salas, Scott C. Weaver

**Affiliations:** a Institute for Translational Sciences, University of Texas Medical Branch, Galveston, TX, USA; b Department of Microbiology and Immunology, University of Texas Medical Branch, Galveston, TX, USA; c Institute for Human Infections and Immunity, University of Texas Medical Branch, Galveston, TX, USA; d UANL-FCB Monterrey, San Nicolás de los Garza, Mexico; e Instituto Nacional de Salud Publica CRISP, Tapachula, Mexico

Zika virus (ZIKV) is an arthropod-borne virus (arbovirus) of the genus *Flavivirus* within the family *Flaviviridae*, originally isolated from the blood of a febrile rhesus macaque in the Ziika forest of Uganda in 1947 [1]. Although serologic evidence indicates that ZIKV has circulated in Africa and Asia for decades [2], only 14 reports of human infection were documented in the literature before 2007 [3]. Subsequently, ZIKV began causing outbreaks on islands in the Pacific before reaching the Americas in 2013 [4]. Beginning in 2015 the virus sparked widespread epidemics in the Americas, garnering international attention due to its association with severe sequelae such as Guillain–Barré syndrome in previously healthy individuals as well as Congenital Zika Syndrome in infants whose mothers were infected during pregnancy [3].

Identification of natural vectors during arboviral outbreaks is critical to both mosquito abatement and public health control strategies. It has been generally accepted that the principal urban vector of ZIKV is the yellow fever mosquito, *Aedes (Stegomyia) aegypti*, although other species have been suggested through both field and experimental studies as having secondary roles (reviewed previously [5]). However, the role of the southern house mosquito *Culex (Culex) quinquefasciatus*, typically the most abundant neotropical urban species, has remained controversial. Only two field-based studies have found any evidence of natural *C. quinquefasciatus* infection by ZIKV [6,7]. However, in both reports, the amount of ZIKV as measured by RNA titres using RT-qPCR was unlikely to be associated with transmission-competence; for example, Guedes et al. [6]. reported Ct values in field-collected pools of *C. quinquefasciatus* pools >37, while the salivary gland pools analysed Elizondo-Quiroga et al. were cell culture-passaged prior to RNA assay [7], eliminating the ability to directly assess transmission potential. However, Guedes et al. correctly pointed out that no quantification of ZIKV titres from *A. aegypti* field collections has been reported for comparison [8].

We previously identified *A. aegypti* as the principal vector during a 2015 ZIKV outbreak in Chiapas State, Mexico by testing mosquito pools derived from collections in and around homes of febrile, tentatively diagnosed patients [9]. *C. quinquefasciatus* collected in similar numbers, as well as *A. (Stegomyia) albopictus*, were not found to be infected. To further assess the principal vector role of *A. aegypti*, we performed real time RT–PCR assays on the mosquito pools described previously and Ct- values were extrapolated to focus-forming unit equivalents per mL (FFUe/mL) via a standard virus titration curve (equation: Titre in FFUe/mL = 2E + 10e^-.696(Ct)^, *R*^2 ^= .988 using ZIKV reference strain Mex1-44, isolated from one of these pools) (Table 1). Of the 155 mosquito pools tested, 15 were found to be unambiguously positive (Ct values of 14.1–26.6) giving a range of 2.2–6.0 log_10_ FFUe/pool. All six pools found to be unambiguously positive and consistent with disseminated, transmission-competence (>10^5^ FFUe/pool) were comprised of *A. aegypti* females only. Four pools were found to be marginally positive at Ct values between 33 and 37, three of which were comprised of *C. quinquefasciatus*, and a single positive pool of *Mansonia titillans* also fell into this ambiguous range. Based on our standard curve, these Ct values correspond to ≈1 FFUe/pool or lower, indicating the amount of virus present was inconsistent with transmission-competence. Although in our experience ZIKV titres extrapolated from real-time PCR Ct values can vary by up to ≈2 fold, depending on the virus strain and infection conditions, in general experimental studies show a typical range of 3.7–5.7 log_10_ FFU/mosquito in the midguts and/or bodies of transmission-competent *A. aegypti* [10,11].

**Table 1.** Results of real-time-quantitative Polymerase chain reaction assays of mosquito pools collected during a Mexican Zika virus outbreak.

Pool ID	Mosquito species	Gender	#/Pool	CT Value	Extrapolated infectious titre from standard curve (FFU/pool)^a^	GenBank Acc. No.
3-7	*A. aegypti*	Female	10	14.12	1.1×10^6^^a^	N/A
1-65		Female	6	14.82	6.6×10^5^^a^	N/A
1-7		Female	6	15.14	5.3×10^5^^a^	KX247632.1
1-71		Female	17	15.472	4.2×10^5^^a^	N/A
1-21		Female	10	16.09	2.7×10^5^^a^	N/A
1-44		Female	1	16.87	1.6×10^5^^a^	KX856011.1
1-19		Female	10	17.84	8.1×10^4^	N/A
1-26		Female	5	18.43	5.4×10^4^	N/A
2-81		Female	1	19.27	3.0×10^4^	KX446590.2
1-69		Female	1	19.52	2.5×10^4^	N/A
1-10		Female	9	19.81	2.1×10^4^	N/A
3-8		Female	10	19.99	1.8×10^4^	N/A
1-58		Female	3	20.32	1.4×10^4^	N/A
1-20		Female	10	22.03	4.4×10^3^	N/A
1-47		Female	1	26.65	1.8×10^2^	N/A
2-43	*M. titillans*	Female	1	33.69	1.3	N/A
2-41	*C. quinquefasciatus*	Female	1	36.92	0.14	N/A
2-36	* *	Female	1	37.01	0.13	N/A
2-38		Female	1	37.45	0.10	N/A

^a^Titres compatible with disseminated, transmission-competent infections, based on previously published laboratory data [10,11].

Although results of experimental vector competence studies are undeniably valuable, such analyses cannot capture the more complex behavioural and ecological considerations that contribute to the more meaningful measurement of vectorial capacity. However, data from the field should be interpreted utilizing context gleaned from experimental studies. Therefore, we report that pools of mosquitos gathered in close proximity to suspected human ZIKV patients during a Mexican outbreak had large amounts of ZIKV RNA present; three of these pools yielded viral isolates (ZIKV strains Mex 1-7, Mex 1-44, and Mex 2-81) that have been widely utilized since 2016 [9]. Vector competence can be highly dependent on the viral strain and geographic origin of the mosquito population tested [5,12], indicating the possibility that the laboratory vector competence findings for *C. quinquefasciatus* represent a specific virus/geographic mosquito strain combination that is conducive for transmission [6]. However, to date, only sequence data from one of these reported *C. quinquefasciatus* derived strains have been deposited into GenBank [13], rendering it difficult for other groups to validate these findings.

Overall, our results validate the principal role of *A. aegypti* as the only mosquito species to be directly incriminated via both experimental ZIKV transmission and surveillance results [5,9,12]. Given the preponderance of evidence in support of *A. aegypti* as the principal ZIKV vector, and lack of direct evidence for a role of *Culex* spp. or other mosquitoes, public health resources should be prioritized for the abatement of this vector.
